# Explaining improvement in diabetes distress: a longitudinal analysis of the predictive relevance of resilience and acceptance in people with type 1 diabetes

**DOI:** 10.1007/s00592-023-02180-2

**Published:** 2023-09-25

**Authors:** Gina Lehmann, Philipp Ziebell, Andreas Schmitt, Bernhard Kulzer, Norbert Hermanns, Dominic Ehrmann

**Affiliations:** 1 Institute of Psychology, Julius-Maximilians-University of Würzburg, Würzburg, Germany; 2 Research Institute of the Diabetes Academy Mergentheim (FIDAM), Johann-Hammer-Str. 24, 97980 Bad Mergentheim, Germany; 3 German Center for Diabetes Research (DZD), Munich-Neuherberg, Germany; 4 Department for Psychology, Otto-Friedrich-University of Bamberg, Bamberg, Germany

**Keywords:** Mental health, Diabetes distress, Psychological resilience, Diabetes acceptance, Prospective study, Type 1 diabetes

## Abstract

**Aims:**

To analyze if midterm improvement in diabetes distress can be explained by resilience, diabetes acceptance, and patient characteristics.

**Methods:**

*N* = 179 adults with type 1 diabetes were enrolled during their stay at a tertiary diabetes center (monocentric enrolment) and followed up over three months in a prospective, observational study (‘DIA-LINK1’). Improvement in diabetes distress was assessed as reduction in the Problem Areas in Diabetes Scale score from baseline to follow-up. Resilience (Resilience Scale-13), acceptance (Diabetes Acceptance Scale), and patient characteristics were analyzed as predictors of improvement in diabetes distress using hierarchical multiple regression.

**Results:**

Greater reductions in diabetes distress were significantly explained by lower diabetes acceptance at baseline (*β* = −0.34, *p* < 0.01), while resilience, diabetes complications, and other person-related variables were not significantly related to changes in diabetes distress (all *p* > 0.05). When change in diabetes acceptance from baseline to follow-up was added to the model, improved diabetes distress was explained by increasing diabetes acceptance (*β* = 0.41, *p* < 0.01) and a shorter duration of diabetes (*β* = -0.18, *p* = 0.03), while baseline diabetes acceptance was no longer significantly associated (*β* = −0.14, *p* > 0.05).

**Conclusions:**

Diabetes acceptance is inversely related to diabetes distress, and increasing acceptance explained greater improvement in diabetes distress. These findings suggest that increasing diabetes acceptance may facilitate the reduction of diabetes distress. Treatment approaches targeting acceptance might be useful for the mental healthcare of people with type 1 diabetes and clinically elevated diabetes distress.

**Supplementary Information:**

The online version contains supplementary material available at 10.1007/s00592-023-02180-2.

## Introduction

Diabetes distress has been in the focus of psychosocial diabetes research for over 25 years [1]. It is one of the most common mental concerns in patients with diabetes and characterized by negative emotional reactions to living with diabetes and its management requirements and impacts on the person’s life [2]. Approximately 30% of people with type 1 diabetes are affected by clinically elevated diabetes distress [1]. Studies suggest that elevated diabetes distress is associated with higher long-term blood glucose levels cross-sectionally and longitudinally [3–5], which may increase the risk for developing serious diabetes complications. Furthermore, elevated diabetes distress has been associated with less optimal diabetes self-care behaviors [4, 6] and impaired quality of life [6]. It is also considered as possible risk factor for comorbid depression in diabetes [5, 7], which is known to be associated with an increased risk for long-term morbidity and mortality in people with diabetes [8, 9].

These negative effects highlight the clinical relevance of diabetes distress and the need for a better understanding its influencing factors and remission. Previous research tended to focus on sources of increased diabetes distress [1]. However, understanding protective factors that may prevent its development or contribute to its reduction are mandatory for the development of prevention and intervention strategies. For instance, resilience is considered as a person’s ability to recover from negative life circumstances and events [10]; it is an established protective factor against mental distress and the development of psychiatric disorders in general as well as across various chronic diseases [11]. This means that people with type 1 diabetes and higher resilience may recover more easily from the diagnosis and from stressful or difficult situations regarding diabetes management and thus, may be less prone to experiencing diabetes distress. Thus, resilience might constitute an important protective factor, as previous research has found higher resilience to be associated with lower diabetes distress [12]. In addition, participation in resilience training programs can yield significant reductions in diabetes distress [13–15].

However, resilience is a comparably broad and stable, trait-like factor [16]. Because diabetes distress relates to specific, individual problem areas, it might be better accounted for by focusing on constructs related to diabetes and its treatment more specifically. Adjusting to living with the chronic condition and its daily demands, impacts, and burdens often implies a process of acceptance and integration of diabetes into one’s daily life as well as one’s self-concept and identity [17]. This process of diabetes acceptance may constitute a lifelong task with people sometimes fluctuating between emotional integration on one side and avoidance or rejection of the condition on the other [18]. Previous research found that lower diabetes acceptance was consistently associated with higher diabetes distress [18, 19].

According to this current evidence, resilience and diabetes acceptance may be decisive factors contributing to the reduction of high diabetes distress. A better understanding of their possible favorable impacts may help improve diabetes care and mental treatments for people with type 1 diabetes. Therefore, this study aimed to analyze the associations between resilience, diabetes acceptance, and the reduction of diabetes distress in people with type 1 diabetes to find potential starting points for interventions to reduce diabetes distress. We analyzed to which extent reductions in diabetes distress over time can be explained by measurements of resilience and diabetes acceptance, while controlling for covariates. Based on the present evidence, we expected that both higher resilience and higher diabetes acceptance might facilitate improvements of diabetes distress.

## Methods

### Study procedures

The data were collected as part of the prospective, observational DIA-LINK Study, which assessed associations between mental factors and health outcomes in people with type 1 diabetes (registered with ClinicalTrials.gov, identifier NCT03811132, and the German Clinical Trial Register (DRKS), identifier DRKS00016593). Recruitment of participants included people with elevated as well as non-elevated diabetes distress and depressive symptoms. Previous work of the DIA-LINK Study addressed the daily experience of diabetes distress [20] and an observed reduced heart rate variability in people with elevated diabetes distress [21]. A detailed description of the study design and procedures is available elsewhere [20]; in brief, 203 participants with type 1 diabetes were enrolled and followed over three months from baseline. Assessments included a baseline questionnaire, a subsequent four-week ambulatory assessment period, and a three-month follow-up questionnaire. The present analysis was based on the baseline and follow-up assessments.

### Participants

Participants were recruited monocentrically at a specialized inpatient center for people with diabetes (Diabetes Center Mergentheim) between 03/2019 and 03/2020. No study-related intervention was provided within the average time of stay (10–12 days); however, all participants were referred to the center for structured diabetes education and multi-professional care to improve their diabetes management as part of their regular stay. This provided treatment was not affected by a person’s decision to participate in the study, so all participants received usual diabetes care at the center. Eligible for participation in the study were people with type 1 diabetes, with a diabetes diagnosis for at least one year, aged between 18 and 70 years, and with sufficient language skills. Persons with severely impaired cognitive function, with somatic or mental disorders that would likely confound the results, with a terminal illness, or being bedridden were excluded. Potentially eligible patients with type 1 diabetes were approached and informed about the study both orally and in writing.

### Variables and measures

Demographic variables (age, sex, years of education) and medical data (diabetes duration, HbA1c, long-term complications like retinopathy, neuropathy, nephropathy, and foot syndrome) were obtained at baseline from medical files, personal interviews, and laboratory assessments. HbA1c was estimated from venous blood samples in the central laboratory of the Diabetes Center Mergentheim (using the Tosoh Automated Glycohemoglobin Analyzer HLC-723G11 meeting IFCC standard; normal range: 4.3–6.1%; 24–43 mmol/mol).

Diabetes distress was assessed using the Problem Areas in Diabetes Scale (PAID) [22]. The questionnaire requests ratings of 20 diabetes-specific emotional problems (e.g., “Feeling scared when you think about living with diabetes?”) on a 5-point Likert scale (from 0– “not a problem” to 4– “serious problem”). The item scores are summed to a total score ranging from 0 to 100 with higher scores suggesting higher diabetes distress. Total scores of 40 or higher are considered as clinically elevated distress [23].

Resilience was assessed with the 13-item Resilience Scale (RS-13) [24, 25], requesting aspects of resilience using self-descriptive statements (e.g., “When I am in a difficult situation, I usually find a way out.”; “I do not let myself be thrown off track so quickly.”). Responses are given on a 7-point Likert scale (from 1– “No, I don’t agree” to 7– “Yes, I agree totally”). The sum score range is 7–91 with higher score indicating higher resilience.

Diabetes acceptance was measured using the short form of the Diabetes Acceptance Scale (DAS), consisting of 10 items regarding the acceptance and integration in one’s life, or rejection and avoidance of diabetes (e.g., “I accept diabetes as part of my life.”; “I avoid dealing with topics related to diabetes.”) [18]. Responses are given on a 4-point Likert scale (from 0– “Never true” to 3– “Always true”). Item scores are summed to a total score between 0–30; higher scores reflect higher diabetes acceptance.

All variables were measured at baseline and re-assessed three months later at follow-up, except for resilience, which was assessed at baseline only, given its relative temporal stability [16].

### Statistical analyses

**Fig. 1:**
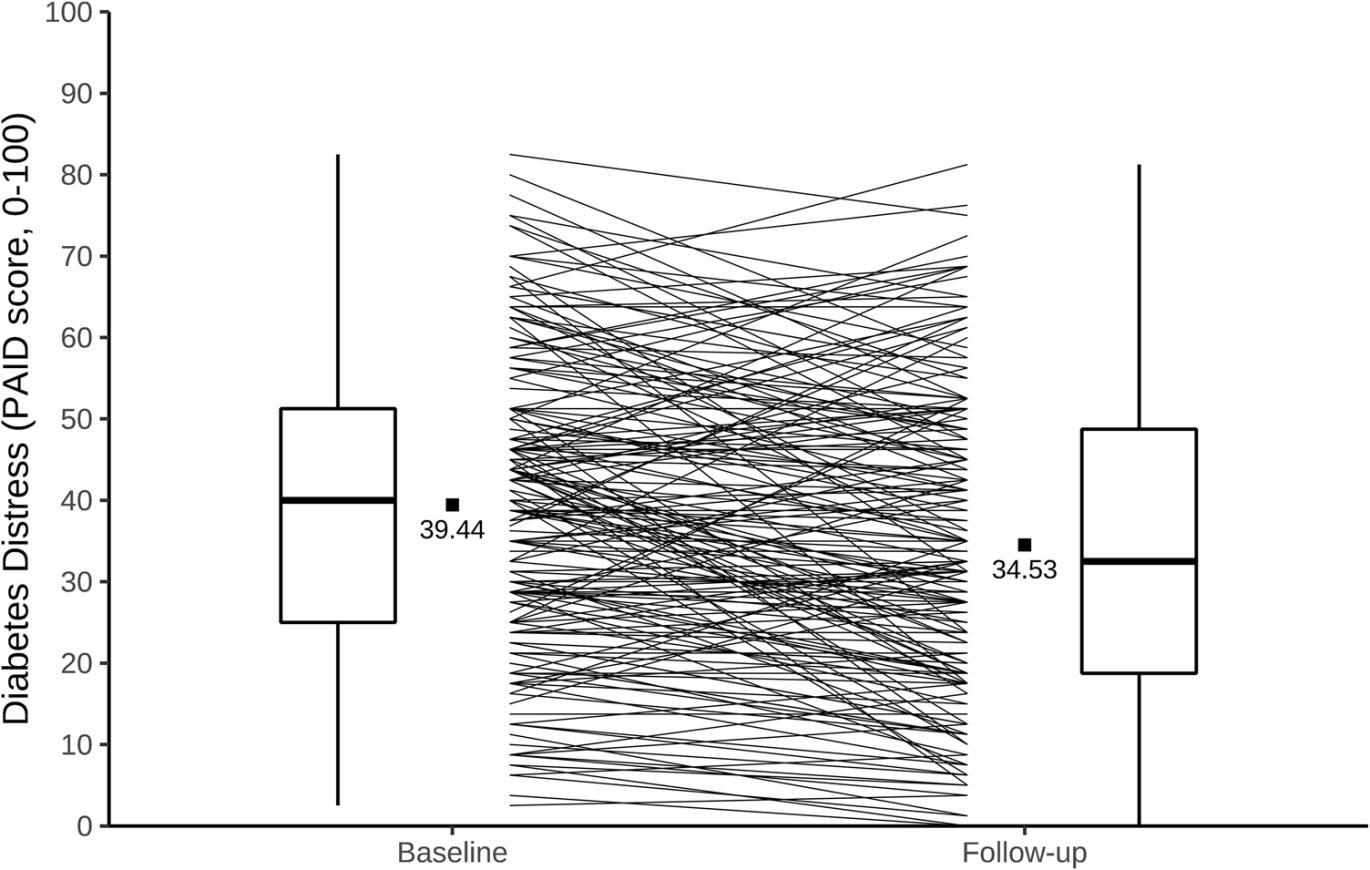
Individual changes in diabetes distress (PAID) from baseline and follow-up. *Note*: Box plots following standard Tukey representations show diabetes distress sum score changes (PAID) from baseline to follow-up. Filled dots represent PAID score means, unfilled dots mark individual PAID sum scores and lines display individual score changes over three months while receiving standard structured diabetes education and multi-professional care. Our analysis revealed a significant difference between the PAID means of baseline versus follow-up (*T*(178) = 4.76, *p* < 0.01), indicating overall improvement

The statistical analyses were performed using IBM SPSS Statistics 27. Figure 1 is produced using R Statistical Software (v4.2.1) [26] with the ggplot2 [27] package. The bivariate associations between all variables were analyzed using Pearson correlations. We used sequential multiple regression analysis to examine whether the reduction of diabetes distress could be explained by resilience and diabetes acceptance, while adjusting for covariates. The reduction of diabetes distress, serving as criterion variable, was operationalized as the difference score between baseline and follow-up assessments with the PAID questionnaire. To estimate independent predictive associations, demographic and clinical variables were included in the regression in the first step (model 1). In the next step, resilience (RS-13 score) was added (model 2). Then, diabetes acceptance (baseline DAS score) was included (model 3). Finally, change in diabetes acceptance from baseline to follow-up (difference of DAS scores) was added to estimate whether increased acceptance would predict reductions of diabetes distress (model 4). The change in diabetes acceptance was calculated by subtracting the baseline DAS score from the DAS score at follow-up, so that higher difference scores reflect greater increases in diabetes acceptance. We decided to focus on the differences of diabetes acceptance between baseline and follow-up to examine changes in diabetes distress due to the daily occupation with the disease over the three months. Demographic covariates were age, sex, education, living in a partner relationship, and being employed. Previous studies found higher diabetes distress associated with a longer diabetes duration [6], higher HbA1c [28], and the presence of long-term complications (neuropathy, nephropathy, retinopathy, and diabetic foot syndrome) [29], so these variables were considered as important covariates. Non-metric variables were dichotomized with 0 for "no" and 1 for "yes". Sex was coded as 0 for “male” and 1 for “female”.

Since diabetes distress is a multifaceted concept and diabetes acceptance may be partly included in its measurement, we additionally analyzed potential content overlap between the questionnaire items using Pearson correlation and regression as a sensitivity analysis.

*P* values < 0.05 (two-tailed) were considered to indicate statistical significance in all analyses.

## Results

### Sample characteristics

Full sample characteristics are displayed in Table 1. A total of 203 people with type 1 diabetes were participated in the DIA-LINK Study. Of those, 179 (88.2%) attended the follow-up study, thus being eligible for the longitudinal analyses. *N* = 103 (57.5%) were women. The mean age was 40.0 ± 12.6 years, and the mean duration of diabetes was 19.5 ± 11.6 years. HbA1c levels were above target with a mean of 8.5 (± 1.7) % (69 (± 18) mmol/mol). *N* = 84 (46.9%) of the participants were diagnosed with one or more long-term complications.

**Table 1:** Descriptive characteristics of the study sample

Variable	Total sample (*N* = 203)	Participants included in the analysis (*n* = 179)
Age (years)	38.6 ± 12.8	40.0 ± 12.6
Female sex	118 (58.1%)	103 (57.5%)
Years of education	13.1 ± 2.6	13.3 ± 2.5
Duration of diabetes (years)	18.5 ± 11.7	19.5 ± 11.6
HbA_1c_ in %	8.7 ± 1.9	8.5 ± 1.7
HbA_1c_ in mmol/mol	71 ± 21	69 ± 18
Diagnosed with any long-term complications +	93 (45.8%)	84 (46.9%)
Diabetic retinopathy	49 (24.1%)	42 (23.5%)
Diabetic neuropathy	66 (32.5%)	61 (34.1%)
Diabetic nephropathy	11 (5.4%)	9 (5.0%)
Diabetic foot syndrome	3 (1.5%)	3 (1.7%)
Diabetes distress at baseline (PAID score, range: 0–100)	40.1 ± 18.2	39.4 ± 18.1
Diabetes acceptance at baseline (DAS score, range: 0–30)	19.2 ± 7.2	19.6 ± 7.1
Resilience at baseline (RS-13 score, range: 7–91)	65.3 ± 12.9	65.9 ± 12.8

Data are means ± SD or *n* (%). + list of diabetes complications: retinopathy, neuropathy, nephropathy, diabetic foot syndrome. *DAS* Diabetes Acceptance Scale, *PAID* Problem Areas in Diabetes Scale, *RS-13* 13-item Resilience Scale

### Changes in diabetes distress from baseline to follow-up

Mean (± *SD*) diabetes distress scores at baseline and follow-up were 39.4 (± 18.1) and 34.5 (± 18.9), respectively. At baseline, 107 persons had PAID scores of 40 and above, suggesting clinically elevated distress; at follow-up, the number was reduced to *N* = 74. Baseline-to-follow-up changes in diabetes distress ranged from -27.5 to 45.0, with a mean (± *SD*) of 4.9 (± 13.8) points on the PAID scale (estimated as baseline minus follow-up). Individual changes in diabetes distress are displayed in Fig. 1. Positive change scores were seen in 63.1% of the participants, reflecting reductions of diabetes distress. Negative scores were seen in 30.2%, indicating increases of distress.

### Bivariate associations of the study variables

Regarding the criterion reduction in diabetes distress, significant associations were found with diabetes distress at baseline (*r* = 0.32; *p* < 0.01), diabetes acceptance at baseline (*r* = -0.27; *p* < 0.01), and the increase in diabetes acceptance (*r* = 0.44; *p* < 0.01). Moderate bivariate associations between the predictors were also found (Table 2).

**Table 2:** Correlations between demographic, clinical, and psychosocial factors

	2	3	4	5	6	7	8	9	10	11
1 Change in diabetes distress (difference of PAID scores)	0.32**	− 0.27**	0.44**	− 0.03	0.03	− 0.03	− 0.07	− 0.12	0.02	0.14
2 Diabetes distress (PAID)	1	− 0.66**	0.17*	− 0.39**	0.03	0.18*	− 0.04	− 0.09	0.06	0.17*
3 Diabetes acceptance (DAS)		1	− 0.44**	0.51**	0.17*	− 0.16*	− 0.04	0.16*	− 0.22**	− 0.01
4 Change in diabetes acceptance (difference of DAS scores)			1	− 0.23**	− 0.04	0.04	0.04	0.01	0.06	0.08
5 Resilience (RS-13)				1	0.00	− 0.22**	0.05	− 0.07	− 0.10	0.08
6 Age					1	0.09	0.10	0.37**	− 0.24**	0.19**
7 Sex						1	0.03	0.02	0.08	− 0.14
8 Years of education							1	0.07	− 0.27**	0.01
9 Duration of diabetes								1	− 0.16*	0.25**
10 HbA1c value									1	0.10
11 Long-term complications of diabetes +										1

Data are Pearson correlations. Indication of two-sided significance: *p < 0.05; **p < 0.01. + list of diabetes complications: retinopathy, neuropathy, nephropathy, diabetic foot syndrome. *DAS* Diabetes Acceptance Scale, *HbA1c* glycated hemoglobin, *PAID* Problem Areas in Diabetes Scale, *RS-13* 13-item Resilience Scale

### Predicting improvements in diabetes distress from baseline to follow-up

Hierarchical regression was conducted through stepwise entry to investigate the impact of resilience and diabetes acceptance on the reduction of diabetes distress, while controlling for demographic and clinical variables at baseline. The assumption of normality was tested via examination of the standard residuals. The P-P-plot suggested a relatively normal distributional shape (with no outliers). No relevant multicollinearity was indicated.

Model 1, including the demographic and clinical variables only, explained 1% of the variance in diabetes distress change (p = 0.28). Variables significantly associated with diabetes distress change were a shorter duration of diabetes (*β* = -0.19, *p* < 0.05) and being diagnosed with diabetic neuropathy (*β* = 0.17, *p* < 0.05).

Model 2, including resilience as additional predictor, explained 1% of variance (*p* = 0.35). Resilience did not significantly predict change in diabetes distress (*β* = 0.02, *p* = 0.84). The *β-*weights of the other predictors did not change notably.

Model 3, adding diabetes acceptance at baseline to the predictors, explained 8% of the variance of diabetes distress change (*p* < 0.05); significantly more than demographic factors and resilience alone (increment *R*^*2*^ = 0.07. *p* < 0.01). Greater reduction of diabetes distress was significantly associated with lower diabetes acceptance at baseline (*β* = -0.34, *p* < 0.01). There were no other significant predictors in this model.

Model 4, additionally assessing changes in diabetes acceptance as predictor of improved diabetes distress, explained 22% of the variance (p < 0.01) (increment *R*^*2*^ = 0.13. *p* < 0.01). While controlling for other variables, only a shorter duration of diabetes (*β* = -0.18, *p* < 0.05) and positive diabetes acceptance change scores (*β* = 0.41, *p* < 0.01) significantly predicted improved diabetes distress at follow-up, indicating that greater increases in diabetes acceptance were associated with greater reductions of diabetes distress. Diabetes acceptance levels at baseline (*β* = -0.14, *p* = 0.14) no longer contributed to the explanation of diabetes distress change. Full results are displayed in Table 3.

**Table 3:** Sequential multiple linear regression of reductions in diabetes distress on demographic and clinical variables, resilience, and diabetes acceptance

	Model 1	Model 2	Model 3	Model 4	
	*R*^*2*^ = 0.01, *F*(11,167) = 1.22 (*p* = 0.28)	*R*^*2*^ = 0.01, *F*(12,166) = 1.12 (*p* = 0.35)	*R*^*2*^ = 0.08*, *F*(13,165) = 2.17 (*p* = 0.01)	*R*^*2*^ = 0.22**, *F*(14,164) = 4.55 (*p* < 0.001)	
Predictors	*β*	*β*	*β*	*β*	*R*^*2*^ (increment)
Model 1: Demographic and clinical factors					*R*^*2*^ = 0.08*, F*(11,167) = 1.22 (*p* = 0.28)
Age	0.06	0.06	0.10	0.10	
Sex	0.06	0.06	0.04	0.06	
Years of education	− 0.09	− 0.09	− 0.13	− 0.13	
Being employed	0.03	0.03	− 0.02	0.01	
Living with a partner	− 0.08	− 0.08	− 0.08	− 0.05	
Duration of diabetes	− 0.19*	− 0.19*	− 0.14	− 0.18*	
HbA1c value	− 0.02	− 0.02	− 0.07	− 0.06	
Diabetic retinopathy	0.01	0.01	0.02	0.02	
Diabetic neuropathy	0.17*	0.17*	0.14	0.13	
Diabetic nephropathy	− 0.03	− 0.03	− 0.03	− 0.04	
Diabetic foot syndrome	0.11	0.11	0.08	0.09	
Model 2: + Resilience					*R*^*2*^ = 0.00,* F*(1,166) = 0.04 (*p* = 0.84)
RS-13 sum score		− 0.02	0.15	0.14	
Model 3: + Diabetes acceptance at baseline					*R*^*2*^ = 0.07**,* F*(1,165) = 13.73 (*p* < 0.001)
DAS sum score			− 0.34**	− 0.14	
Model 4: + Increase in diabetes acceptance					*R*^*2*^ = 0.13**,* F*(1,164) = 30.54 (*p* < 0.001)
Difference in DAS sum score (baseline to FU)				0.41**	

Data are standardized regression weights (*β*). Indication of two-sided significance: *p < 0.05; **p < 0.01. *DAS* Diabetes Acceptance Scale, *FU* follow-up, *HbA1c* glycated hemoglobin, *RS-13* 13-item Resilience Scale

### Sensitivity analyses

To estimate potential construct confounding and to support the results from the preceding calculations, potential overlap of the constructs diabetes acceptance and diabetes distress and their corresponding items was additionally analyzed.

Correlations of the DAS items with the PAID total score showed the highest correlations for items 7 ("Diabetes contributes to me being dissatisfied with my life") and 8 ("Having diabetes makes me sad/depressed"), in line with the items’ overlapping contents with diabetes distress as measured by the PAID.

To exclude the possibility that the weights of our regression analysis were exaggerated due to this possible overlap, we repeated the multiple regression analysis using diabetes acceptance sum scores excluding the items 7 and 8 of the DAS. The adjusted *R*^2^ and weights (*β*) were not notably different from the results above (see Appendix), supporting that our findings are unlikely to be confounded by content overlap.

## Discussion

This study investigated potential positive impacts of psychological resilience and diabetes acceptance on diabetes distress reduction in people with type 1 diabetes using data from the prospective, observational DIA-LINK Study.

Diabetes distress, resilience, and diabetes acceptance were significantly associated in the bivariate analysis. These results are in line with previous findings that both resilience as well as lower diabetes acceptance levels were cross-sectionally associated with more severe diabetes distress [12, 19], suggesting that people with higher resilience and higher diabetes acceptance are more likely to report lower diabetes distress.

The regression analysis showed that diabetes acceptance at baseline was a significant predictor of diabetes distress reductions. Individuals with less diabetes acceptance at baseline showed greater changes in diabetes distress than people with higher acceptance levels at baseline. This may be due to the fact that people with lower diabetes acceptance at baseline also had higher diabetes distress and thus had higher room for improvement in both diabetes acceptance and diabetes distress. Indeed, the change in diabetes acceptance significantly contributed to a diabetes distress reduction, indicating that greater increases in diabetes acceptance were associated with greater reductions of diabetes distress. This suggests that people whose diabetes acceptance improved over the course of three-months experienced less diabetes distress at follow-up. After adding change of diabetes acceptance, baseline differences in diabetes acceptance no longer predicted diabetes distress change, suggesting that the process of improving diabetes acceptance is a stronger predictor than the general level diabetes acceptance.

Roth et al. [16] suggested that resilience as a personality trait protects against different forms of life distress. Contrary to expectations, resilience was not prospectively associated with a distress reduction. Although people with greater resilience may be less prone to experiences of high diabetes distress, those who do experience high distress despite more resilience may be unlikely to achieve improvement in diabetes distress based on resilience alone, at least not on short term.

Among the covariates, only a shorter duration of diabetes was consistently associated with diabetes distress change. Possibly because recently diagnosed people with diabetes were less adapted to their chronic disease at baseline and thus had higher diabetes distress values at this time of measurement [1]. These values were then more likely to be improved than lower diabetes distress in better adapted individuals. In addition, people with a shorter duration of diabetes may be more motivated in improving their diabetes related issues and coping with feelings of distress during the daily occupation with the chronic disease over the three months.

Since some items of the PAID and DAS questionnaire show a relevant content overlap, which might confound associations between the constructs diabetes distress and diabetes acceptance, a sensitivity analysis excluding the items with shared contents was performed. This analysis showed consistent results with the original regression, supporting that these results are valid and may be generalized.

In summary, when all variables were included in the model, change in diabetes acceptance was independently associated with improvement in diabetes distress. This suggests that low diabetes acceptance might play a role in the persistence of increased diabetes distress, while increasing acceptance may help lowering diabetes distress. This supports the consideration of diabetes acceptance in treatment. Diabetes acceptance could be a promising starting point within healthcare approaches aiming to support people with elevated diabetes distress. This assumption is in line with prior findings that acceptance-oriented interventions such as Acceptance and Commitment Therapy can counteract negative effects of non-acceptance on diabetes distress [30–32].

Several limitations of this study must be considered. First, the number of study participants included in our analyses and the length of follow-up was limited due to the sampling strategy and aims of the DIA-LINK Study as well as missing data in some participants. Second, the sample was collected at a specialized diabetes center whose population may not represent the primary care setting. Furthermore, the questionnaire survey method is subject to biases such as social desirability or retrospective memory bias. Especially regarding the resilience construct, which has been operationalized differently in previous studies, different operationalizations might provide different results [33]. Last, the interactions between the analyzed parameters may be complex and causal inferences cannot be drawn due to the non-experimental study design, thus the presented findings must be interpreted with care. Future experimental studies can provide more information on questions such as to which extent diabetes acceptance can directly cause improvements in diabetes distress and might thus be promising for the design of specifically tailored interventions to support individuals with negative developments in diabetes distress. Strengths of this study include its prospective design and the multivariate adjustment of the analyses, allowing the identification of independent predictors of improvement in diabetes distress over time.

## Conclusion

Increasing diabetes acceptance is linked to decreasing diabetes distress. Given these findings, future research on diabetes distress merits including the less explored concept of diabetes acceptance to gain a more complete picture of diabetes distress and underlying relationships and mechanisms.

### Supplementary Information

Below is the link to the electronic supplementary material.

Supplementary file1 (PDF 142 kb)
