# Supplementary material for: Explaining improvement in diabetes distress: a longitudinal analysis of the predictive relevance of resilience and acceptance in people with type 1 diabetes
Source: Acta Diabetol. 2023 Sep 25;61(2):151–9. doi: 10.1007/s00592-023-02180-2 (PMC10866794; doi:10.1007/s00592-023-02180-2)
Supplement: Supplementary file 1 — Supplementary file1 (PDF 142 kb) [file 592_2023_2180_MOESM1_ESM.pdf]

Acta Diabetologica

Explaining improvement in diabetes distress: a longitudinal analysis of the predictive relevance of resilience and acceptance in people with type 1 diabetes

Gina Lehmann (BSc)[a], Philipp Ziebell (MSc)[a], Andreas Schmitt (PhD)[b,c], Bernhard Kulzer (PhD)[b,c,d], Norbert Hermanns (PhD)[b,c,d], Dominic Ehrmann (PhD)[b,c,d]

[a] Julius-Maximilians-University of Würzburg, Institute of Psychology, Würzburg, Germany

[b] Research Institute of the Diabetes Academy Mergentheim (FIDAM), Bad Mergentheim, Germany

[c] German Center for Diabetes Research (DZD), München-Neuherberg, Germany

[d] Otto-Friedrich-University of Bamberg, Department for Psychology, Bamberg, Germany

Corresponding Author: PD Dr. Dominic Ehrmann, Research Institute Diabetes Academy Mergentheim (FIDAM), Johann-Hammer-Str. 24, 97980 Bad Mergentheim, Germany. Email: ehrmann@fidam.de

Supplementary Table A1. Sequential multiple linear regression of reductions in diabetes distress on demographic and clinical variables, resilience, and diabetes acceptance using diabetes acceptance sum scores excluding the items 7 and 8 of the DAS

	**Model 1**	**Model 2**	**Model 3**	**Model 4**	
	R^2=0.01, F(11,167)=1.22 (p=0.28)	R^2=0.01, F(12,166)=1.12 (p=0.35)	R^2=0.08*, F(13,165)=2.17 (p=0.01)	R^2=0.15**, F(14,164)=4.55 (p<0.001)	
**Predictors**	***β***	***β***	***β***	***β***	**R^2 (increment)**
**Model 1: Demographic and clinical factors**					R^2=0.08, F(11,167)=1.22 (p=0.28)
Age	0.06	0.06	0.10	0.09	
Sex	0.06	0.06	0.05	0.07	
Years of education	-0.09	-0.09	-0.13	-0.12	
Being employed	0.03	0.03	-0.03	0.00	
Living with a partner	-0.08	-0.08	-0.07	-0.05	
Duration of diabetes	-0.19*	-0.19*	-0.15	-0.17*	
HbA1c value	-0.02	-0.02	-0.08	-0.07	
Diabetic retinopathy	0.01	0.01	0.02	0.01	
Diabetic neuropathy	0.17*	0.17*	0.14	0.14	
Diabetic nephropathy	-0.03	-0.03	-0.03	-0.03	
Diabetic foot syndrome	0.11	0.11	0.09	0.10	
**Model 2: + Resilience**					R^2=0.00, F(1,166)=0.04 (p=0.84)
RS-13 sum score		-0.02	0.15	0.16	
**Model 3: + Diabetes acceptance at baseline**					R^2=0.08**, F(1,165)=13.73 (p<0.001)
DAS sum score			-0.34**	-0.2*	
**Model 4: + Increase in diabetes acceptance**					R^2=0.07**, F(1,164)=30.54 (p<0.001)
Difference in DAS sum score (baseline to FU)				0.30**	

Note: Data are standardized regression weights (β). Indication of two-sided significance: * $p < 0.05$, ** $p < 0.01$. DAS = Diabetes Acceptance Scale; FU = follow-up; HbA1c = glycated hemoglobin; RS-13 = 13-item Resilience Scale.
